# Encouraging brisk walking with the free Active10 app in postnatal women who had a hypertensive pregnancy: “Just Walk It” feasibility study

**DOI:** 10.1371/journal.pone.0282066

**Published:** 2023-02-21

**Authors:** Mohammad S. Razai, Bonnie Trinder, Alice Perry, Matthew Cauldwell, Fiona Reid, Pippa Oakeshott

**Affiliations:** 1 Population Health Research Institute, St George’s University of London, London, United Kingdom; 2 St George’s University Hospital NHS Trust, London, United Kingdom; 3 Kings College London, London, United Kingdom; University of Campinas, BRAZIL

## Abstract

**Objective:**

To explore the feasibility of a future trial to investigate whether encouraging use of the free NHS smartphone app Active10 increases brisk walking and reduces blood pressure (BP) in postnatal mothers who had hypertensive disorders of pregnancy (HDP).

**Design:**

3-month feasibility study.

**Setting:**

London maternity unit.

**Population:**

21 women with HDP.

**Methods:**

At recruitment we recorded initial (booking) clinic BP and asked participants to complete a questionnaire. Two months after delivery all participants were sent (by post/email/WhatsApp) a “Just Walk It” leaflet encouraging them to download the Active10 app and walk briskly for at least 10 minutes/day. This was backed by a telephone call after 2-weeks. Assessments were repeated 3-months later, and included telephone interviews about the acceptability and use of Active10.

**Main outcome measures:**

Were recruitment rate, follow-up rate and acceptability/use of Active10.

**Results:**

Of 28 women approached, 21 (75%, 95% CI 55.1–89.3%) agreed to participate. Age range was 21–46 years and five (24%) self-identified as black ethnicity. One woman dropped out of the study, and one became ill. The remaining participants (90%, 19/21, 95% CI 69.6–98.8%) were followed up after 3-months. Ninety-five percent (18/19) downloaded the Active10 app and 74% (14/19) continued using it at 3-months, averaging 27-minutes brisk walking/day according to Active10 weekly screenshots. Comments included: “Brilliant app”, “Really motivates me”. Mean BP was 130/81mmHg at booking and 124/80mmHg at 3-months follow-up.

**Conclusions:**

The Active10 app was acceptable to postnatal women after HDP and may have increased minutes of brisk walking. A future trial could explore whether this simple, low-cost intervention could reduce long-term BP in this vulnerable group.

## Introduction

Recent evidence has highlighted the importance of promoting physical activity in pregnant and postnatal women, particularly during the COVID-19 pandemic, with its adverse impacts on physical and mental health [1, 2]. Although we know that using apps can increase physical activity [3–5], we lack robust data on the effectiveness of apps in promoting physical activity in postnatal women who have had high blood pressure (BP) during pregnancy (known as hypertensive disorders of pregnancy or HDP).

Each year in England, 70,000 women have HDP [6]. Women of black ethnicity and older mothers are particularly vulnerable [7, 8]. Compared with women with normal BP during pregnancy, women who have HDP have four times the long-term risk of developing high BP and double the risk of future heart attack, stroke, or death due to cardiovascular disease [6, 9].

A cohort study of 55,000 nurses suggested the risk of hypertension following HDP might be reduced by adopting beneficial lifestyles such as doing regular moderate to vigorous physical activity (MVPA) [10]. However, preliminary investigation of a UK GP database (Clinical Practice Research Datalink) found that only one in ten of 1375 women who had had HDP, had advice about exercise documented in their medical records in the year after delivery.

Regular brisk walking at 3-4mph (i.e., MVPA) starting in the postnatal period might reduce the risk of developing high blood pressure in women who had HDP. MVPA can reduce systolic BP (SBP) by 4-9mm Hg in people with mild hypertension and 1-2mm Hg in normotensives [11]. The free NHS Active 10 app, developed by Public Health England, encourages at least 10 minutes brisk walking every day [12, 13]. The app includes goal setting, feedback and encouragement to support behaviour change. Our preliminary PPI (Patient and Public Involvement) work in 39 postnatal women in primary are showed that most found Active10 acceptable, especially when backed up by a leaflet and/or reminder telephone call encouraging them to use it [14, 15].

Although widely used and promoted across the NHS, the Active10 app has never been evaluated in a trial. There are also increasing numbers of physical activity apps for pregnant and postnatal women, but they tend to be of low quality with poor perceived outcome, and few align with current evidence-based guidelines on physical activity [16, 17].

We aimed to conduct a 3-months feasibility study of giving postnatal women who had had HDP a “Just walk it” leaflet [14, 15] encouraging use of the Active10 app, backed by a telephone reminder after 2-weeks. (The leaflet was co-designed with our PPI group of six ethnically diverse women who had had a hypertensive pregnancy. Please see S2 Fig.) We hypothesised that this intervention would increase daily step count and reduce BP in women who had HDP.

## Methods

### Ethics approval

Ethics approval was granted by Health Research Authority and Health and Care Research Wales (HCRW) on 27^th^ May 2020. REC reference: 20/LO/0693. Written informed consent was obtained from all participants in the study.

### Participants

**Fig 1:**
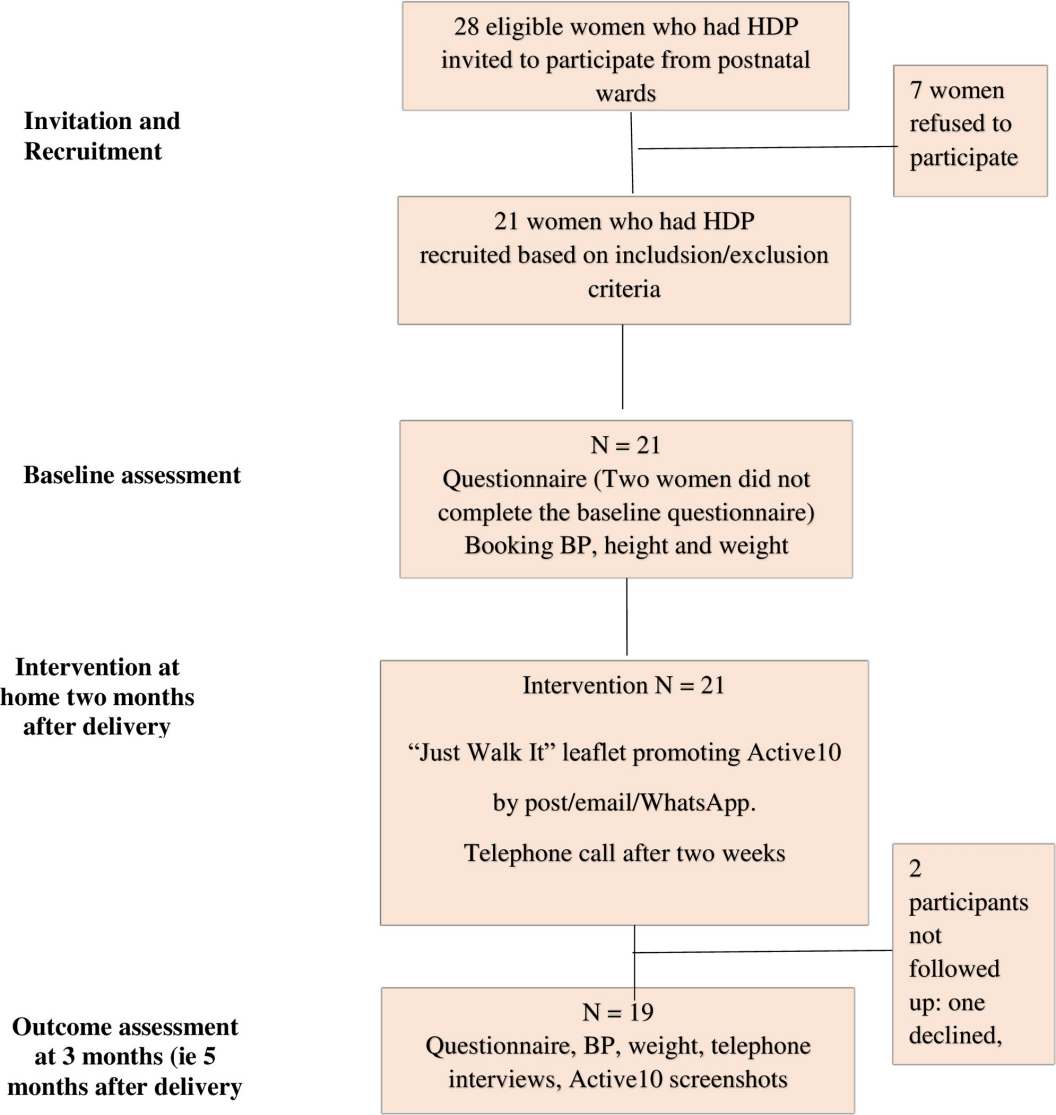
“Just Walk It” feasibility trial flow chart: Use of a brief advice leaflet and the Active10 app to increase brisk walking in mothers who had hypertensive disorders of pregnancy (HDP).

**Fig 1** shows the flow of participants. Between October and December 2020, consecutive women with HDP on the St George’s hospital’s antenatal and postnatal wards and delivery suite were approached by a midwife or researcher and asked if they were willing to take part in a study encouraging regular brisk walking after HDP. Women who were interested were given a patient information leaflet and encouraged to ask questions.

### Inclusion criteria were

Aged ≥18 years, ability to consent, diagnosed with HDP confirmed by an obstetrician in hospital records, between 28 weeks gestation and 2 weeks postpartum, lives within 10 miles of the hospital, owns a smart phone.

### Exclusion criteria were

Participant already meeting Department of Health physical activity guidelines (150 minutes of MVPA or 75 minutes of vigorous activity weekly using the validated question: “As a rule do you do at least half an hour of moderate or vigorous exercise such as walking or sport on five or more days a week?”), difficulty walking such as using a walking aid, midwives consider the patient too unwell to be approached by researchers.

Inclusion and exclusion criteria were assessed before consent using advice from the midwives and/or obstetricians, patients’ medical notes, and asking potential participants directly.

### Baseline assessment

Eligible women who were willing to take part completed a paper consent form. They were asked to complete a brief non-validated questionnaire **(S1 Fig)** on an electronic device/tablet (i.e., iPad) or on paper, including demographic details, medical history, their estimated baseline walking minutes per week (none, ≤10 minutes, >10 minutes), as well as frequency of brisk walking per week (none, 1–3 times, 4–6 times, ≥7 times) and minutes spent walking on one of those days, dietary information and smoking status. The questionnaire also asked participants about their self-efficacy for brisk walking in the postnatal period i.e., did they think they would manage to walk briskly? Baseline BP and weight were extracted from initial (booking) clinic records. (In the UK, the booking clinic assessment is usually conducted by midwives at around 10 weeks gestation). Due to COVID-19 pandemic restrictions and time pressures (i.e., physical distancing measures and reduced visiting hours for non-essential staff), five women did not complete the baseline questionnaire at the time of recruitment of whom three completed it up to 4-weeks later by phone/email.

### Intervention

Two months after delivery, all participants were sent (by post, email and/or WhatsApp based on women’s preferred method of contact as indicated in the consent form) a “Just Walk It” leaflet (S2 **Fig**) providing information on the possible benefits of brisk walking after HDP and encouraging them to download the Active10 app and walk briskly for at least 10 minutes per day. This was accompanied by a telephone call from MSR after 2 weeks to ask if the woman had downloaded or used the app and reminding her about follow-up after 3 months. If she had used Active10, MSR asked the woman to share her brisk walking activity from the app by sending an anonymised screenshot which had the app activity without containing any identifiable information about the participant (**S3 Fig**). If the woman had not downloaded or used the app, she was encouraged to try it and a further call arranged after 2 weeks.

### Outcome assessment

Assessments were repeated after 3 months (i.e., 5 months after delivery, **S4 Fig)** by email or telephone call. We also asked participants for the most recent measurements of their postnatal BP and weight since leaving hospital. This could be from their own BP monitor and scales if available, or from measurements done in GP or hospital clinics [18]. Where the participant did not have a record of her BP or weight, we asked her GP to provide the most recent postnatal measurements.

### Outcome measures

1. Recruitment rate: number of women who agreed to participate as a percentage of all invited eligible women.
2. Follow-up rate at 3 months post-intervention: number of women followed up after 3 months as a percentage of those recruited.
3. Information on BP and weight at 3 months post-intervention (5 months post-delivery)
4. Acceptability and use of Active10 (from questionnaires, mobile phone Active10 screenshots (**S3 Fig**) and telephone interviews at 3 months).
  - How many women downloaded the Active10 app
  - How many women used it to monitor their brisk walking
  - How many achieved at least 10 minutes of brisk walking in a day, at 3 months post-intervention–from a screenshot of participants’ Active10 record over the past 7 days
  - Women’s opinions of Active10

### Sample size and statistical analysis

We aimed to recruit around 40 participants to this feasibility study in line with Teare et al. [19], but recruitment had to be curtailed due to COVID-19. Data were entered and analysed using Microsoft Excel. We summarised descriptive data on minutes of brisk walking, BP and weight using means and standard deviations. Recruitment and follow-up rates are provided with exact 95% confidence intervals using PASS v15 software. (PASS 15 Power Analysis and Sample Size Software (2017). NCSS, LLC. Kaysville, Utah, USA, ncss.com/software/pass.)

## Results

### Recruitment

Recruitment took place from October to December 2020, in those weeks (about 10 out of 13) when recruitment was possible due to COVID-19. There were usually up to three potentially eligible women with HDP on the maternity wards each week. Of 28 eligible women approached, 21 took part in the study, a response rate of 75% (21/28; 95% CI 55.1 to 89.3%). Of the seven women who declined to participate, five mentioned ‘lack of time’ and two stated they were ‘not interested’. Fourteen women requested the leaflet by email and 9 requested by WhatsApp or text (some requested both by email and WhatsApp/text).

### Characteristics of participants

Table 1 presents the baseline characteristics of the participants. Their mean age was 33 years (range 21–46). A third of participants (7/21) were from ethnic minorities including five women (24%) of black ethnicity. Over half of participants (12/19, 63%) had been educated to degree level, and most (74%) knew that women who have had HDP are at increased risk of cardiovascular disease in future. A quarter (26%) said they felt down or depressed, and only around half (53%) felt confident that they would be able to walk briskly for 10 minutes a day on 5 days a week starting around two months after delivery.

**Table 1:** Demographic and clinical characteristics of 21 participants.

Characteristics	Value
Age in years (n = 21), mean (range)	33 (21–46)
Ethnicity (n = 21)	
Black	5 (24%)
White	14 (66%)
Asian	1 (5%)
Mixed	1 (5%)
Qualifications (n = 19)	
None	1 (5%)
GCSE/O Levels	3 (16%)
BTEC/NVQ	3 (16%)
Bachelors degree	11 (58%)
Masters degree	1 (5%)
Comorbidities (n = 19)	
Hypertension	17 (89%)
Pre-eclampsia	8 (42%)
Depression	5 (26%)
Anxiety	3 (16%)
Smoking/Vaping (n = 19)	0 (0%)
BMI at booking (kg/m^2^): (n = 19) mean (range)	26.1 (18.3–34.6)
Blood Pressure (mmHg): (n = 19) mean (SD)	
Baseline at booking clinic	Systolic 129.6 (14.1)
	Diastolic 80.7 (12.7)
Anti-hypertensive medications at recruitment (n = 19)	
Yes	9 (47%)
Alcohol (units/week) (n = 19)	
None	12 (63%)
1 to 4	1 (5%)
5 to 14	6 (31%)
Fruits (portions/day) (n = 19)	
1 to 4	16 (84%)
5 or more	3 (16%)
Vegetables (per plate) (n = 19)	
One quarter or less	11 (58%)
Half	6 (31%)
More than half	2 (10%)
Salt at the table (n = 19)	
Never	2 (10%)
Rarely	1 (5%)
Sometimes	6 (32%)
Often	5 (26%)
Always	5 (26%)
Walking per week baseline: No. (%) (n = 19)	
None	2 (10%)
Up to 10 minutes	2 (10%)
More than 10 minutes	15 (79%)
Brisk walking per week baseline: No. (%) (n = 19)	
None	10 (52%)
1 to 3 times	4 (21%)
4 to 6 times	2 (10%)
7 times or more	3 (16%)
Use of digital app to monitor walking at baseline (n = 19)	
Yes	3 (15%)
No	16 (85%)
Awareness of increased long-term cardiovascular risk (n = 19)	
Yes	14 (74%)
No	5 (26%)
How sure to walk briskly 10 minutes per day 5 times per week (n = 19)	
Very sure	10 (53%)
Pretty sure	6 (31%)
A little sure	3 (16%)
Depression screening questions (over the last month) n = 19	
Feeling down, depressed or hopeless	5 (26%)
Little interest or pleasure in doing things	3 (16%)

### Follow-up rates

One woman dropped out after 4 weeks because of low mood. Another woman became severely unwell after 3 weeks and was therefore deemed unsuitable for further follow-up (in both cases this was unrelated to the study). The follow up rate at 3 months (i.e. 5 months post-partum) was 90% (19/21; 95% CI 69.6–98.8%), of whom 74% (14/19) provided information on minutes of brisk walking (by sending anonymised screenshots of Active10 app activity by email or WhatsApp). Fifteen women (79%) also provided data on their most recent postnatal BP taken at their general practice or at home (if self-monitoring).

### Brisk walking and Active10 app use after 3 months

Table 2 shows outcomes at 3 months. Most of the followed-up women (95%, 18/19) downloaded the Active10 app. (The woman who did not download the app had an unwell baby still in hospital at 3 months follow up.) Neither of the two women who dropped out had downloaded the app by three weeks. Six women downloaded the app after the second follow-up call at 4 weeks.

**Table 2:** Outcomes at three months in 19 women followed up.

Downloaded the Active10 app	18/19 (95%)
Used the Active10 app to monitor brisk walking	14/19 (74%)
Achieved at least 10 minutes brisk walking in a day in past 5 days	12/19 (63%)
Brisk walking minutes per day in 14 women: mean (range)	27 (2–96)
Total walking minutes per day in 14 women: mean (range)	60 (8–127)
BP mmHg in 15 women: mean (SD)	
Systolic	124.4 (12.1)
Diastolic	79.9 (6.5)
On anti-hypertensive medications at 3 months No (%)	3/19 (16%)
On non-antihypertensive medications at 3 months No (%)	5/19 (26%)
BMI kg/m^2^ in 18 women: mean (range)	25.4 (18.4–34.3)

After 3 months, 14 women (74%) were still using the app regularly to monitor their brisk walking. Screenshots showed that the mean of the average time over the last 7 days spent brisk walking by these women was 27.8 minutes per day (range 2.4 to 96.2 minutes), and that 11 of the 19 followed-up women (58%) had done at least 10 minutes brisk walking daily for at least 5 of the past 7 days.

### Acceptability-women’s opinions of the Active10 app

Most women found the app very helpful, especially its ability to motivate, its real-time feedback and its ease of use.

Comments at 3 months included:

‘I will keep using Active10 app, it really motivates me.’ *Black African 28 years*

‘I have downloaded the app and been using it for a couple of months. It’s very user friendly as the app does everything for you! I like the design and colours. I also like the alerts reminding me to go walking. I also like that it gives you a round up each month so you can see how active you are being.’ *White 42 years*

‘It’s a brilliant app, I walk brisk 17–20 minutes most days and a lot of normal walking too, I check the app every day, it tells me how many minutes are brisk. The app is good for anytime but especially after pregnancy, it pushes you a little more to be active and lose weight. I need to lose a few more kilos and also will be good for my blood pressure.’ *White 46 years*

‘I’ve downloaded the app and have been going on walks which is good. It’s nice to get out of the house!’ *White 25 years*

‘I use it a lot! It is such a good app, it gives feedback. I will continue to use it.’ *White 24 years*

‘I found the app easy and useful overall when I am using it.’ *Black African 33 years*

‘I think the app is great in that I don’t have to remember to turn it on every day, it automatically begins reading.’ *Black Caribbean 42 years*

However, not all comments were positive. One participant reported that she could not do any brisk walking due to feeling unwell. In addition, some women may not have realised that they did not walk fast enough (@ 3-4mph) to be recorded as walking briskly by the app. For example, the Active10 screenshot for the past week for one participant showed she had walked for 499 minutes in total, but only 76 minutes were recorded as brisk walking. This may have led to misunderstandings:

‘I think it’s not accurate enough because it gives me shorter durations for my daily brisk walking.’ Black African 42 years

‘I am walking from my home to the station every day. Unfortunately, it doesn’t record it.’ Asian 40 years

### Exploratory data on BP and weight at 3 months follow up

Table 2 shows that mean BP (in 15 women with follow up data; 4 from GP and 11 from home monitoring) had fallen from 130.0/81.0 mmHg SD (14.1/12.7) at baseline in the booking clinic to 124.4/79.9 mmHg SD (12.0/6.5) at follow-up. Mean BMI (in 18 women) was 26.1 kg/m^2^ at baseline and 25.4 kg/m^2^ at 3 months. Only 3 of the 19 women (16%) remained on antihypertensive medication.

## Discussion

### Main findings

The Active10 app was acceptable to postnatal women who had HDP. Almost three quarters of those followed up were still using it to monitor their brisk walking 5 months after delivery. These women had a mean of 27 minutes for their average daily MVPA in the past week. The recruitment rate was 75% and follow up after 3 months was 90%.

### Strengths and limitations

This is the first study promoting the Active10 app in postnatal women with HDP. It had good recruitment and follow-up rates and included a diverse group of women, including 24% of black ethnicity, a group particularly vulnerable to HDP, hypertension and stroke. All the women who tried succeeded in downloading the app. This included seven women from ethnic minority groups and suggests that introducing this technology may be unlikely to increase ethnic health inequalities (Fuller evaluation of the impact of this app on ethnic and socioeconomic health inequalities is out of the scope of this feasibility study.) Using mobile phones, WhatsApp, email and post, we were able to conduct both the intervention and follow up remotely and in line with COVID-19 recommendations. We did not have to exclude any participant due to not possessing a mobile phone. We also showed that most women who had had HDP, some of whom had been quite ill, could still manage to fit brisk walking into their daily routines in the postnatal period.

The main limitation is that this was a small study based at a single centre and with recruitment curtailed by COVID-19 visiting restrictions and staff shortages. Although we included a diverse population group, we had a high proportion of older participants with degree level qualifications which may not be representative of the general population. We had screen shot data on minutes of brisk walking, but we did not have objective data such as accelerometery. In addition, due to COVID-19 we were unable to visit participants at home to obtain reliable, standardised baseline and follow up BP and weight. Instead, we had to rely on booking clinic measurements at baseline, and GP records or home BP monitoring at 3 months follow up. Thus methods and site of BP measurement varied, and BP was not usually assessed at the same time as the questionnaire was completed. Despite having had HDP, two patients did not have a follow-up blood pressure check in the community, highlighting the potentially fragmented nature of postnatal care after HDP. Finally, we only had data after 3 months follow up, so we cannot be sure that any increase in MVPA will persist longer term.

### Interpretation in the light of other evidence

Our findings support previous reports that exercise apps are generally acceptable, feasible to use and associated with an increase in MVPA [10, 13]. Further, the study also supports our previous pilot and PPI work [14, 15] showing that many postnatal women find Active10 very motivating and user friendly. Most women were interested in receiving advice about brisk walking and in one study 60% (9/15) downloaded the Active10 app [15]. However, there is a paucity of studies of the effect of community-based postnatal interventions on BP after HDP. In the Oxford SNAP-HT trial of self-management of postnatal hypertension [20], BP in control women was 130.8/85.4 mmHg at recruitment and 126.6/85.8 mmHg at 3 months follow up. This is broadly consistent with our findings. All participants in SNAP-HT were on antihypertensives at recruitment suggesting they may have had more severe HDP.

## Conclusions

This feasibility trial is the first use of the Active10 app to increase brisk walking in postnatal women after HDP. Using a brief advice leaflet and a free walking app, we have shown that a simple, cheap and acceptable physical activity intervention has the potential to increase brisk walking in postnatal women after HDP. Our recruitment and follow-up rates (despite the challenges of COVID-19) suggest a definitive randomised controlled clinical trial is feasible. The recent post-covid move back to many more face-to-face consultations could allow more reliable assessments of baseline and follow-up BP and weight in a future study, perhaps done by researchers during a home visit. Given the long-term risk of potentially fatal cardiovascular diseases associated with HDP, an increase in moderate to vigorous physical activity by brisk walking using an app could have widespread public health relevance. Further research is needed to see if the apparent increase in physical activity seen in our study in the postnatal period can be confirmed and sustained in the longer term and leads to a reduction in blood pressure and cardiovascular risk.

## Supporting information

S1 Checklist STROBE statement—checklist of items that should be included in reports of observational studies. (DOCX)

S1 Fig Confidential baseline questionnaire. (DOCX)

S2 Fig “Just Walk It” leaflet co-designed with PPI group and emailed/Whatsapp/posted to participants two months after delivery. (DOCX)

S3 Fig Screenshot of Active10 app activity. (DOCX)

S4 Fig Final questionnaire at 12 weeks (by text, email or telephone). (DOCX)
