# Supplementary figures and images for: Encouraging brisk walking with the free Active10 app in postnatal women who had a hypertensive pregnancy: “Just Walk It” feasibility study
Source: PLoS One. 2023 Feb 21;18(2):e0282066. doi: 10.1371/journal.pone.0282066 (PMC9942986; doi:10.1371/journal.pone.0282066)

**
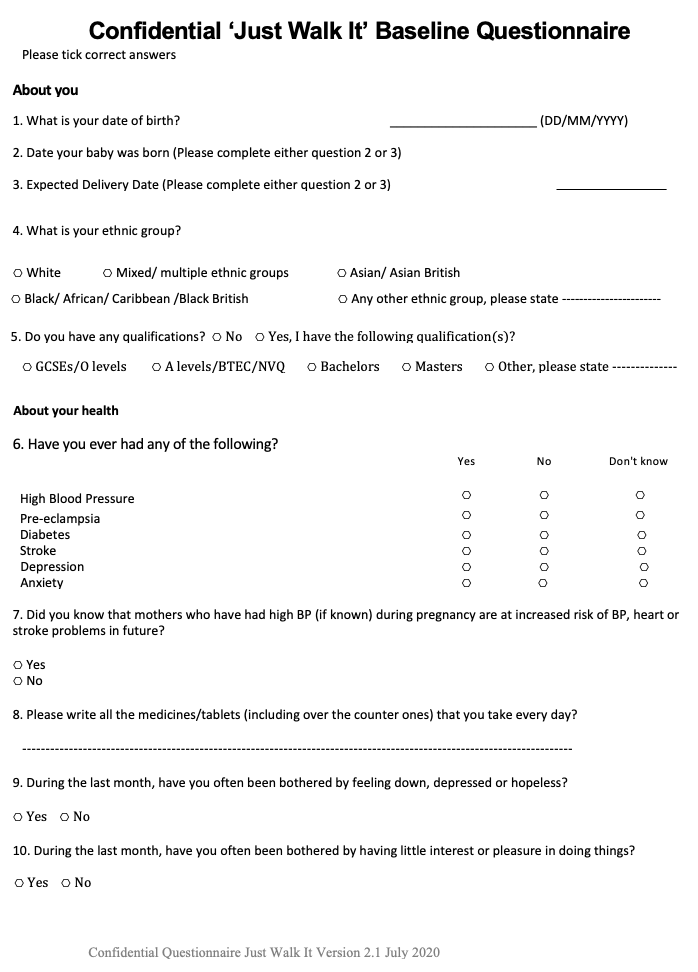
**

**S1 Fig. Confidential baseline questionnaire**

**
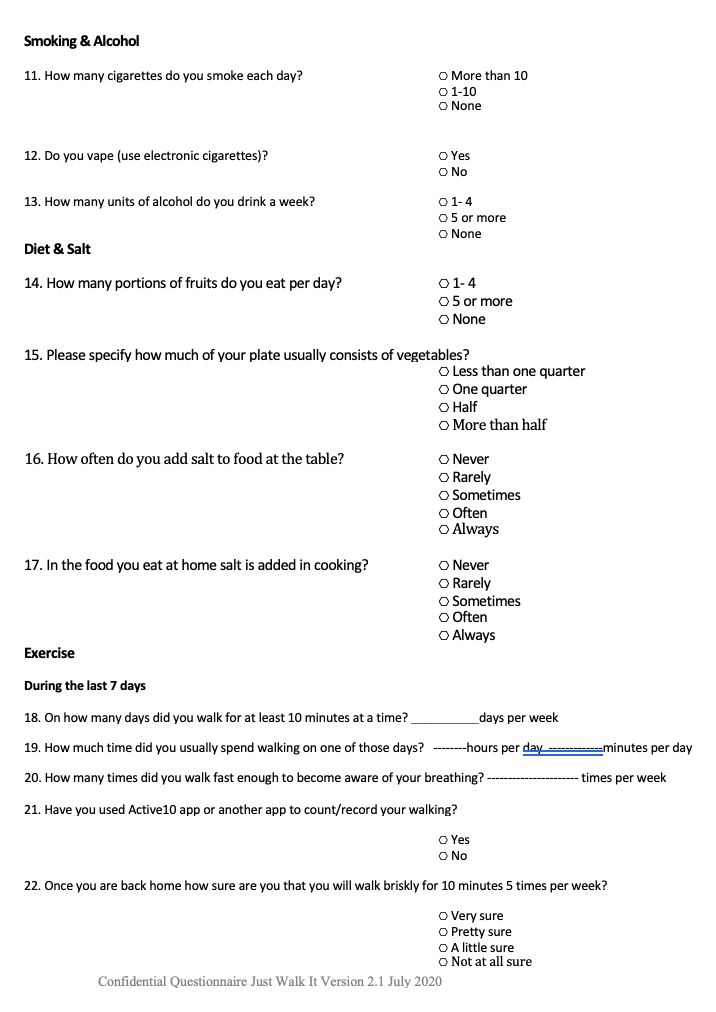
**

Supplement: S1 Fig — (DOCX) [file pone.0282066.s002.docx]

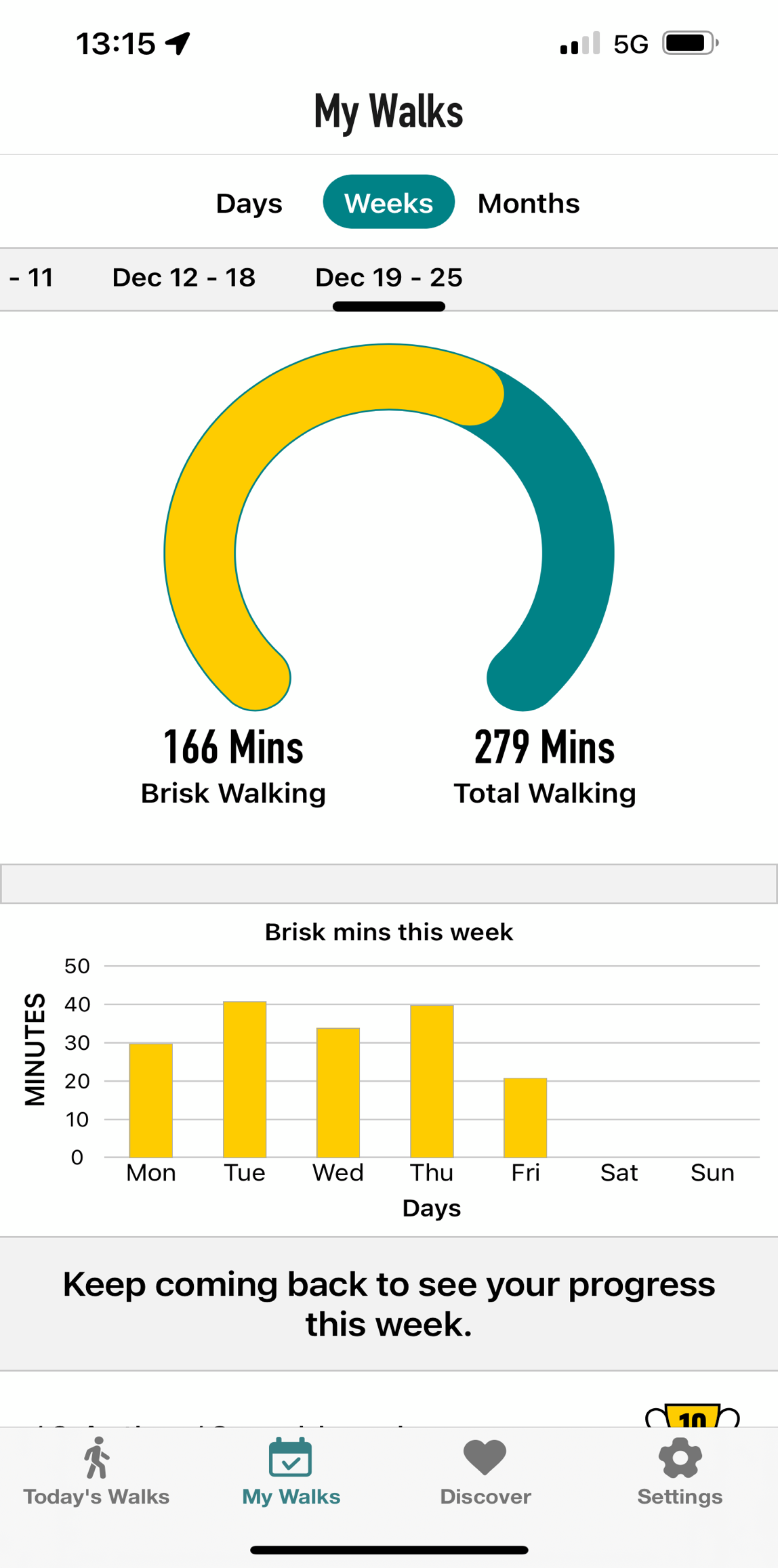


**S3 Fig. Screenshot of Active10 app activity**

Supplement: S3 Fig — (DOCX) [file pone.0282066.s004.docx]
