# Supplementary material for: Encouraging brisk walking with the free Active10 app in postnatal women who had a hypertensive pregnancy: “Just Walk It” feasibility study
Source: PLoS One. 2023 Feb 21;18(2):e0282066. doi: 10.1371/journal.pone.0282066 (PMC9942986; doi:10.1371/journal.pone.0282066)
Supplement: S2 Fig — (DOCX) [file pone.0282066.s003.docx]

**
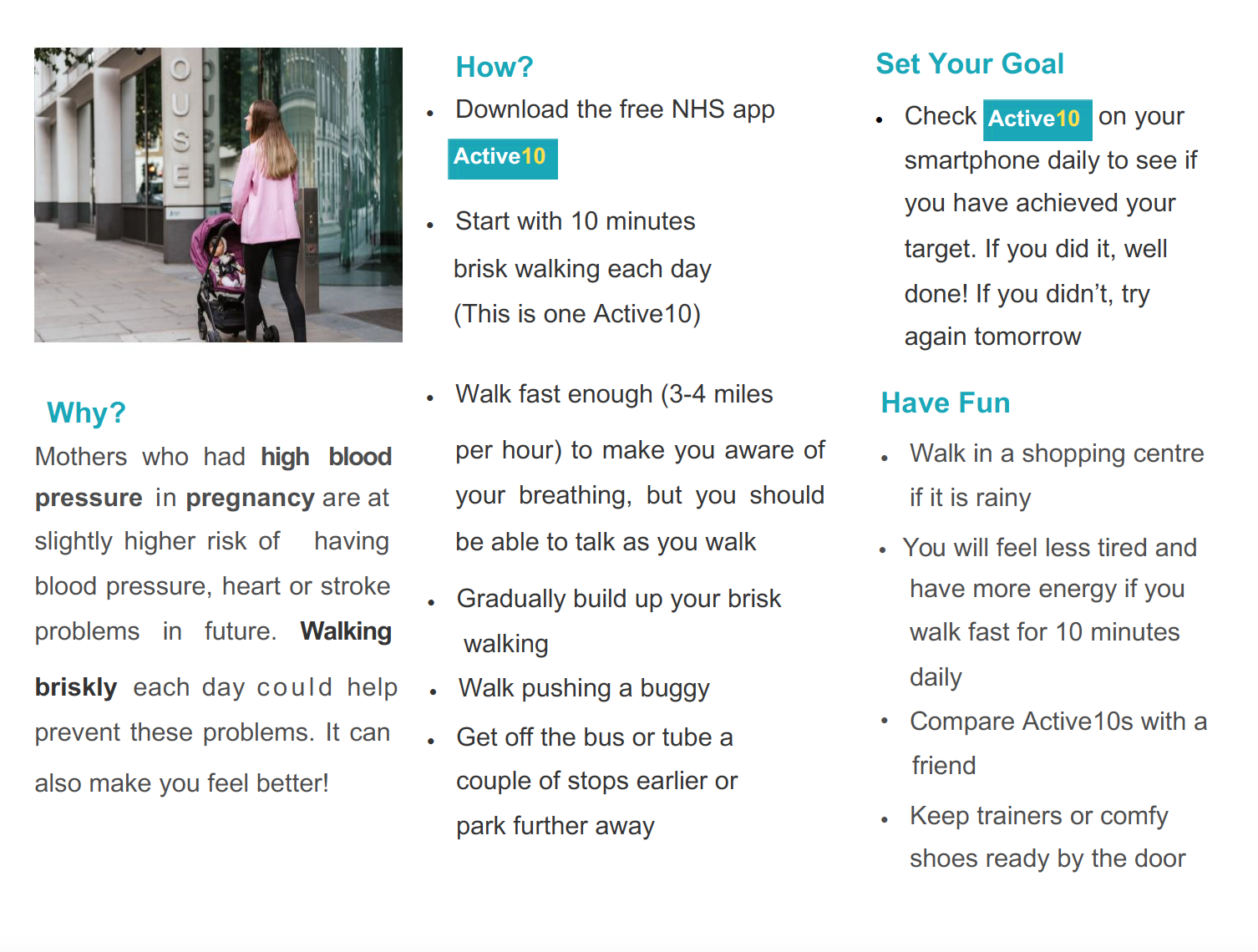

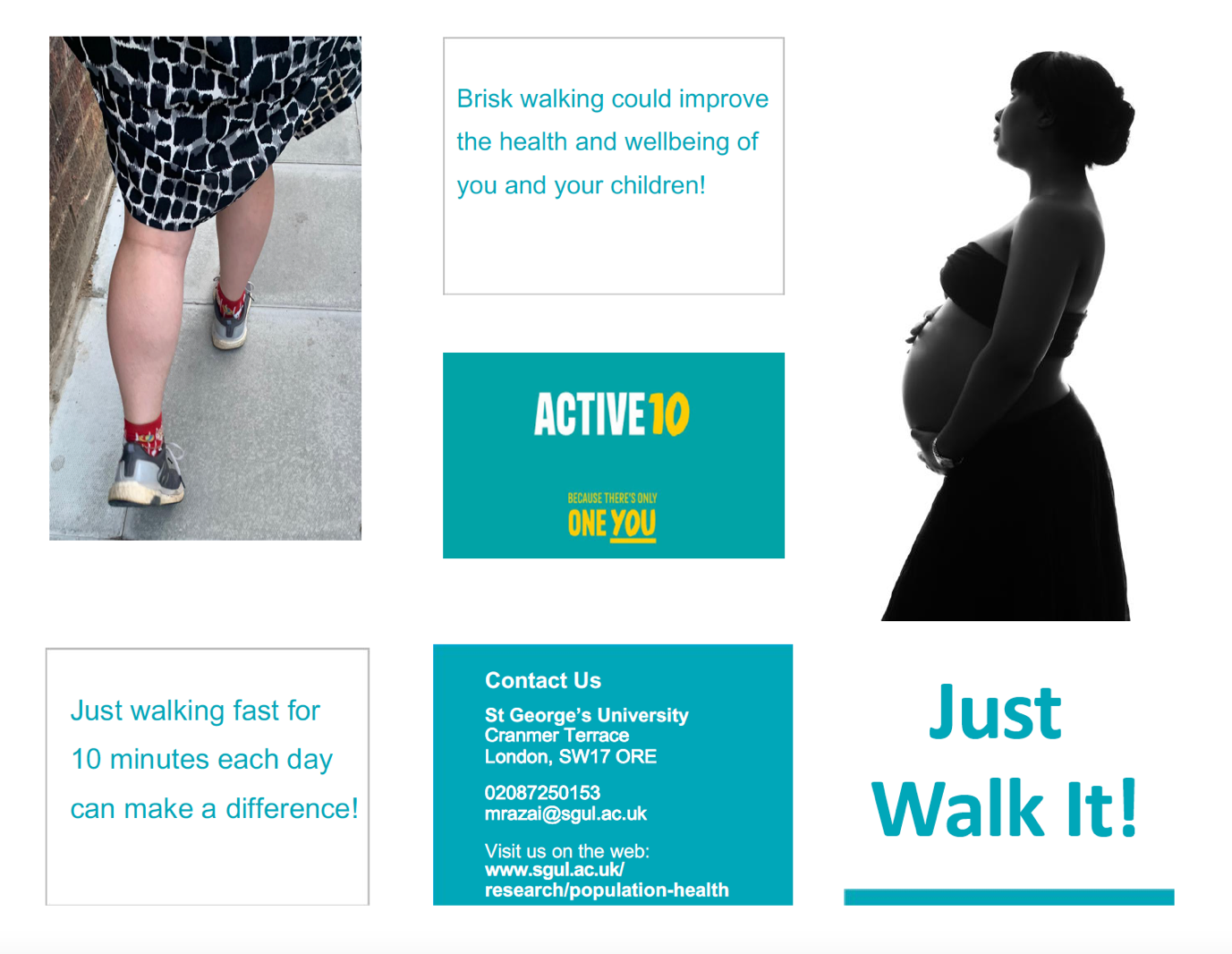
**

**S2 Fig. “Just Walk It” leaflet co-designed with PPI group and emailed/Whatsapp/posted to participants two months after delivery**
