# Supplementary material for: Encouraging brisk walking with the free Active10 app in postnatal women who had a hypertensive pregnancy: “Just Walk It” feasibility study
Source: PLoS One. 2023 Feb 21;18(2):e0282066. doi: 10.1371/journal.pone.0282066 (PMC9942986; doi:10.1371/journal.pone.0282066)
Supplement: S4 Fig — (DOCX) [file pone.0282066.s005.docx]

**S4 Fig. Final questionnaire at 12 weeks (by text, email or telephone)**

1. Have you downloaded the Active10 app?
2. Have you used the Active10 app?
3. How are you finding the Active10 app?
4. Will you continue to use the Active10 app?
5. How many minutes of brisk walking have you managed in one day/ one week? Please kindly send screenshot of walking and brisk walking in the past week
6. If you have not downloaded Active10 please can you tell me the reason
7. Can you tell me your most recent blood pressure reading since you came home with your baby? When was it taken?
8. Can you tell me your weight?
9. Any other comments?
